# Comparison between visit-to-visit office and 24-h blood pressure variability in treated hypertensive patients

**DOI:** 10.1097/HJH.0000000000003582

**Published:** 2023-10-09

**Authors:** Giuseppe Mancia, Rita Facchetti, Fosca Quarti-Trevano, Raffaella Dell’Oro, Cesare Cuspidi, Guido Grassi

**Affiliations:** a University Milano-Bicocca; b Clinica Medica, Department of Medicine, University of Milano-Bicocca, Milan, Italy

**Keywords:** ambulatory blood pressure, antihypertensive treatment, office blood pressure, visit-to-visit blood pressure variability

## Abstract

**Objectives:**

In any treated hypertensive patient office blood pressure (BP) values may differ between visits and this variability (V) has an adverse prognostic impact. However, little information is available on visit-to-visit 24-h BPV.

**Methods:**

In 1114 hypertensives of the ELSA and PHYLLIS trials we compared visit-to-visit office and 24-h mean BPV by coefficient of variation (CV) of the mean systolic (S) and diastolic (D) BP obtained from yearly measurements during a 3–4 year treatment period. Visit-to-visit BPV during daytime and night-time were also compared.

**Results:**

Twenty-four-hour SBP-CV was about 20% less than office SBP-CV (*P* < 0.0001). SBP-CV was considerably greater for the night-time than for the daytime period (20%, *P* < 0.0001). Results were similar for DBP and in males and females, older and younger patients, patients under different antihypertensive drugs or with different baseline or achieved BP values. In the group as a whole and in subgroups there was significant correlations between office and 24-h BP-CV but the correlation coefficients was weak, indicating that office SBP or DBP CV accounted for only about 1–4% of 24-h SBP or DBP-CV values.

**Conclusion:**

Twenty-four-hour mean BP across visits is more stable than across visit office BP. Visit-to-visit office and 24-h BPV are significantly related to each other, but correlation coefficients are low, making visit-to-visit office BP variations poorly predictive of the concomitant 24-h BP variations and thus of on-treatment ambulatory BP stability.

## Graphical abstract

**Figure:**
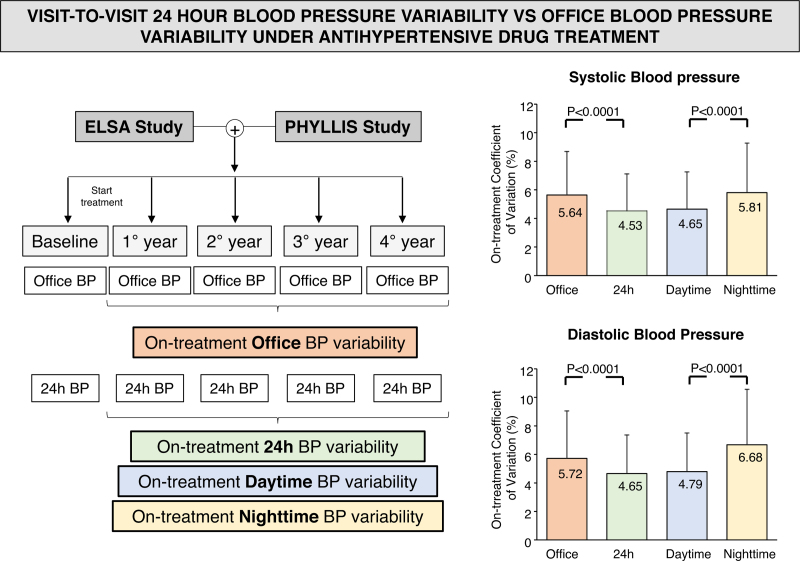

## INTRODUCTION

Post-hoc analyses of outcome based randomized trials have shown that in treated hypertensive patients the greater is the number of visits in which office blood pressure (BP) is controlled, the lower is the incidence of cardiovascular events and mortality even when data are adjusted for the mean office BP throughout the treatment period [1–3]. It has also been shown that a similar relationship exists between cardiovascular outcomes and office BP variations between visits, independently on the on-treatment mean office BP values [4–6]. This has established the importance for an elevated office BP to be consistently controlled by treatment [1]. It has also established the importance for office BP values collected during treatment to be as uniform as possible through the treatment period.

Despite the documented prognostic value of ambulatory BP [7–11] little evidence exists on visit-to-visit variability of 24-h mean BP, because outcome trials have never been based on 24-h BP [12], which has usually been measured only once during treatment in a small and nonrandomized fraction of the recruited patients. To-date some information on this issue has been made available only by the European Lacidipine Study on Atherosclerosis (ELSA) [13], in which both office and 24-h BP were measured in treated hypertensive patients at yearly intervals for 4 years. In this study 24-h mean BP was reported to vary between visits less than office BP [14] but the data analysis did not address visit-to-visit 24-h BPV in clinically important patient subgroups. Furthermore, no information was provided on visit-to-visit BPV during different periods of the 24 h, including the night-time period during which BP has an especially important prognostic value [9–12]. Mindful of the documented prognostic importance of ambulatory BP [7–11] the aim of the present study was to provide a comprehensive analysis of the extent and characteristics of visit-to-visit 24 h as well as day and night-time BPV in treated hypertensive patients and compare them with those obtained by office BP. Data were retrieved from both the ELSA and the Plaque Hypertension Lipid-Lowering Italian Study (PHYLLIS), i.e. the only other study in which multiple yearly office and ambulatory BP measurements were obtained during prolonged antihypertensive drug treatment [15,16]. Because in ELSA treatment made use of a calcium channel blocker or a beta-blocker while in PHYLLIS the antihypertensive agents were an angiotensin converting enzyme inhibitor or a diuretic, data analysis was extended to visit-to-visit 24-h mean BP according to the antihypertensive treatment type. It was further extended to different demographic (sex and age) subgroups as well as to subgroups with different baseline or on-treatment office and 24-h BP values

## METHODS

The design and methods of the ELSA and PHYLLIS studies have been described in detail elsewhere [13,15]. Briefly, ELSA was a prospective, multicenter, randomized, double-blind trial comparing the effects of atenolol (50 to 100 mg once daily) and lacidipine (4 to 6 mg once daily) on the progression of carotid artery intima-media thickness in mild-to-moderate hypertensive patients aged 45–75 years. Open label hydrochlorothiazide (12.5–25 mg once daily) was added if after a three month titration of the initial drugs BP was not lowered to <140/90 mmHg. PHYLLIS was also a prospective, multicenter, randomized, double-blind trial comparing the effects of treatment on carotid intima-media thickness of moderate hypertensive and hypercholesterolemic patients. Treatment consisted of hydrochlorothiazide (25 mg once daily) or fosinopril (20 mg once daily) with or without a background statin (simvastatin, 20 mg once daily). A calcium channel blocker was added if, after a titration period of 3 months, BP was not <140/90 mmHg. Treatment was up-titrated during the first 3 months, after which it remained stable for about 4 years (ELSA) or 3 years (PHYLLIS) [13,15].

### Office BP measurements

In both studies office BP was measured by a mercury sphygmomanometer at baseline, at monthly intervals during the treatment titration and at 6 months intervals after the 6th month. The first and fifth Korotkoff sounds were taken to indicate systolic (S) and diastolic (D) BP values, respectively. At each visit three measurements were obtained with the patient in the sitting position for at least 5 min. The average of the three values was used as the representative value for the visit [14,16].

### Ambulatory BP measurements

In both studies 24-h BP was measured at baseline and at yearly intervals during treatment, within a maximum of 1 week after measurement of office BP [14,16]. According to the trial protocols only monitoring devices validated by studies based on international protocols were used [17]. In all patients of the ELSA trial and in most patients of the Phyllis trial the device used was Spacelabs 90207 (Spacelabs Inc., Redmond, Washington, USA) [14,16]. The BP monitoring began in the morning, after assumption of the study drugs, and lasted 24 h. The monitoring devices were programmed to provide automatic measurements every 15 min during the day (0600 h to midnight) and every 20 min during the night (midnight to 0600 h). Patients were instructed to undergo their usual activities during the monitoring period, to keep the arm extended and immobile during the cuff inflations and to return the following morning for the device removal. The recordings were analyzed centrally and considered for data analysis only if according to prespecified criteria, valid BP readings were ≥70% of the expected readings (*n* = 92) and at least one valid reading per hour was available for ≥21 h [14,16].

### Data analysis

SBP and DBP office, 24-h, day and night mean values were calculated for each patient, using the values obtained from the yearly on-treatment visits. In each patient and for each BP (office, 24-h daytime and night-time) calculation was made of the standard deviation (SD) of the mean SBP or DBP throughout the treatment period. The SD was divided by the mean SBP or DBP value and multiplied by 100 to obtain for each BP the coefficient of variations (CV), which was taken as the measure of the intraindividual visit-to-visit SBP or DBP variability. The SBP or DBP-CV has been shown to have no association with the mean SBP or DBP value [6], which makes it an independent measure of BPV. Visit-to-visit office 24-h, daytime and night-time BPV were calculated also in (i) males and females (ii) patients aged less than and greater than the median age of the entire patient group (iii) differently treated patients (iv) patients with a baseline BP or an average on-treatment BP less than or greater than the median value of the analyzed group. Calculation was also made of the number of visits in which BP was controlled by treatment, the values indicating control being those mentioned by hypertension guidelines, i.e. <140/90, <130/80, <135/85, and <120/70 mmHg for office, 24-h, daytime and night-time, respectively [12,18]. Only patients with three or four valid yearly ambulatory BP monitoring during the treatment period were included in the analysis and compared with the corresponding yearly office BP data. Data from the two trials were pooled and comparisons between office and ambulatory BP values were made by the t-test for paired observations. The McNemar's test was used to compare the number of controlled office and 24-h BP. ANOVA with the Bonferroni correction was used to compare BPV among different treatments. Ambulatory and office BP were correlated via the Pearson correlation coefficient. The square of the correlation coefficient (*r*^2^) was used to calculate the 24-h SBP-CV or DBP-CV accounted for by office SBP-CV or DBP-CV values. When written after mean values the symbol ± refers to the SD of the mean. A *P* <0.05 was taken as the level of statistical significance.

## RESULTS

A total of 1114 patients (943 from ELSA and 171 from PHYLLIS) met the criteria for quantification of visit-to-visit office, 24-h, day and night SBP and DBP variability. In these patients, the number of valid ambulatory SBP values was close to the expected one for every year of treatment (Table S1, Supplemental Data). As shown by the baseline data of Table 1, the average age of the patients was about 56 years, and there was a slight prevalence of males. Office SBP and DBP were both in the hypertension range and this was the case also for 24-h, daytime and night-time BP values. Serum cholesterol and triglycerides were elevated, whereas serum glucose and creatinine values were normal. As also shown by Table 1, treatment was accompanied by a clearcut reduction in office, 24-h, daytime and night-time SBP and DBP values. There was also a small but significant reduction of office and ambulatory heart rate together with small alterations of serum lipid and glucose. Carotid intima-media thickness was above the normal values at baseline [13,19] and did not show any appreciable change during treatment.

**TABLE 1:** Baseline and on-treatment demographic and clinical values in patients from ELSA and PHYLLIS combined

Variable	Baseline	On-treatment	*P*-value
*N*	1114		
Male prevalence (%)	53.1		
Age (years)	56.3 ± 7.3		
Systolic BP, office (mmHg)	162.8 ± 11.5	141.1 ± 10.6	<0.0001
Diastolic BP, office (mmHg)	100.6 ± 4.8	85 ± 5.3	<0.0001
Heart rate, office (beats/min)	75.4 ± 9	70.9 ± 8.9	<0.0001
Systolic BP, 24-h (mmHg)	140.3 ± 13.6	131.6 ± 11	<0.0001
Diastolic BP, 24-h (mmHg)	87.4 ± 9.2	80.6 ± 7.6	<0.0001
Heart rate, 24-h (beats/min)	74 ± 8.7	69.8 ± 9.4	<0.0001
Systolic BP, daytime (mmHg)	144 ± 14	134.4 ± 11.1	<0.0001
Diastolic BP, daytime (mmHg)	90.5 ± 9.4	83.1 ± 7.9	<0.0001
Heart rate, daytime (beats/min)	76.6 ± 9.2	71.9 ± 10.1	<0.0001
Systolic BP, night-time (mmHg)	127.1 ± 14.7	121.2 ± 12.4	<0.0001
Diastolic BP, night-time (mmHg)	76.3 ± 10	71.4 ± 8.2	<0.0001
Heart rate, night-time (beats/min)	64.8 ± 8.4	62 ± 7.8	<0.0001
Serum cholesterol (mg/dl)	230.2 ± 38.9	228.5 ± 40.9	0.1091
Serum HDL cholesterol (mg/dl)	52.2 ± 15.9	53.4 ± 16.1	0.0601
Serum triglycerides (mg/dl)	134.1 ± 64.6	142.2 ± 79.2	<0.0001
Serum creatinine (mg/dl)	0.96 ± 0.18	0.96 ± 0.19	0.2757
Serum Glucose (mg/dl)	95.9 ± 18.4	101.8 ± 28.7	<0.0001
Carotid IMT (mm)	1.17 ± 0.23	1.18 ± 0.22	<0.0001

Baseline means and standard deviation from combined ELSA and PHYLLIS data. On-treatment values have been obtained by averaging the mean of available yearly data during the whole treatment period (4 years for ELSA and 3 years for PHYLLIS). BP, blood pressure; IMT, intima-media thickness; HDL, high density lipoprotein.

### Office and ambulatory BP control

**FIGURE 1:**
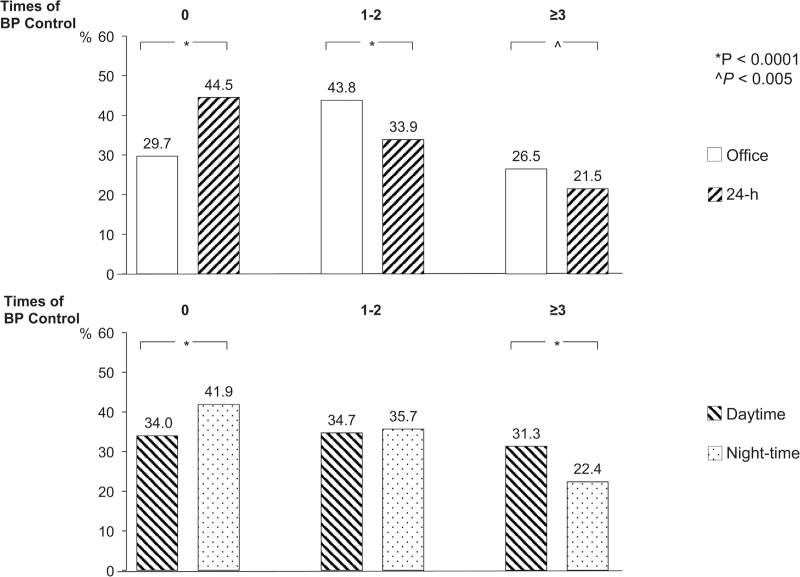
Percentage (%) of patients never achieving blood pressure (BP) control (0 visits), achieving partial BP control (1–2 visits) or achieving BP control in all or almost all visits (≥3 visits) during the yearly visits planned by ELSA and PHYLLIS during the treatment period. Data are shown for office vs. 24-h BP control (top panel) and daytime vs. night-time BP control (bottom panel). BP values indicating control are taken from international hypertension guidelines. Symbols as in Table 1.

Figure 1, top panel, shows that office BP was never controlled (no visit with BP <140/90 mmHg) in 29.7% the patients and that persistent or almost persistent office BP control throughout the treatment period (3–4 visits) occurred in 26.5% of the patients. Compared to office BP control, patients in whom 24-h BP was never controlled (no visit with <130/80 mmHg) were, significantly more (44.5%) whereas patients in whom 24-h BP control was persistently controlled were significantly less (21.5%) As shown in Fig. 1, bottom panel, patients who never achieved nighttime BP control were significantly more frequent than patients who did not achieve daytime BP control (41.9% vs. 34.0%) whereas the opposite was the case for persistent night-time vs. daytime BP control (22.4% vs. 31.3%).

### Visit-to-visit on-treatment blood pressure variability

**FIGURE 2:**
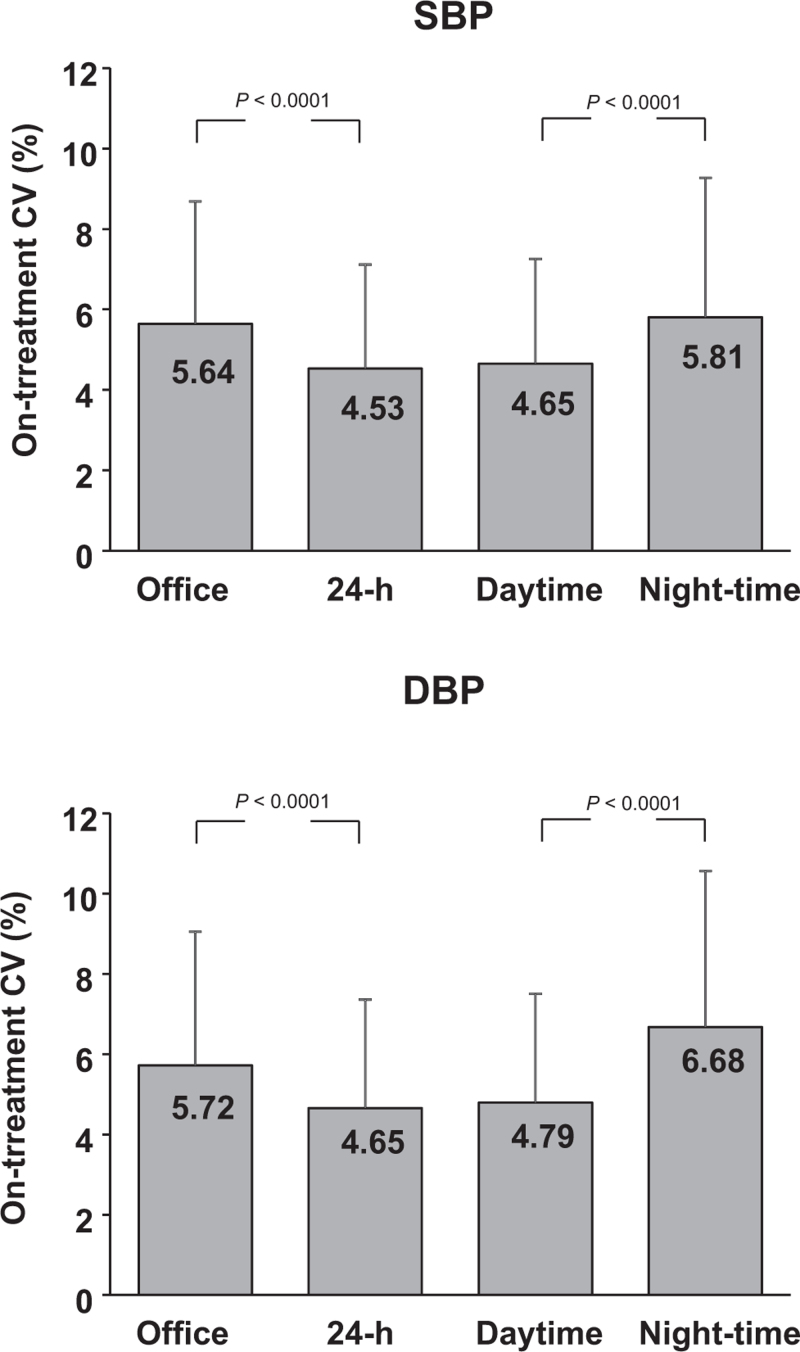
On-treatment visit-to-visit office, mean 24-h, mean daytime and mean night-time SBP and DBP variability in 1114 patients with ≥ 3 yearly visits. Data are shown as means ± standard deviation (SD). Variability is quantified as the coefficient of variation (CV) of the mean BP values throughout the treatment period. Symbols as in Table 1 and Fig. 1.

**FIGURE 3:**
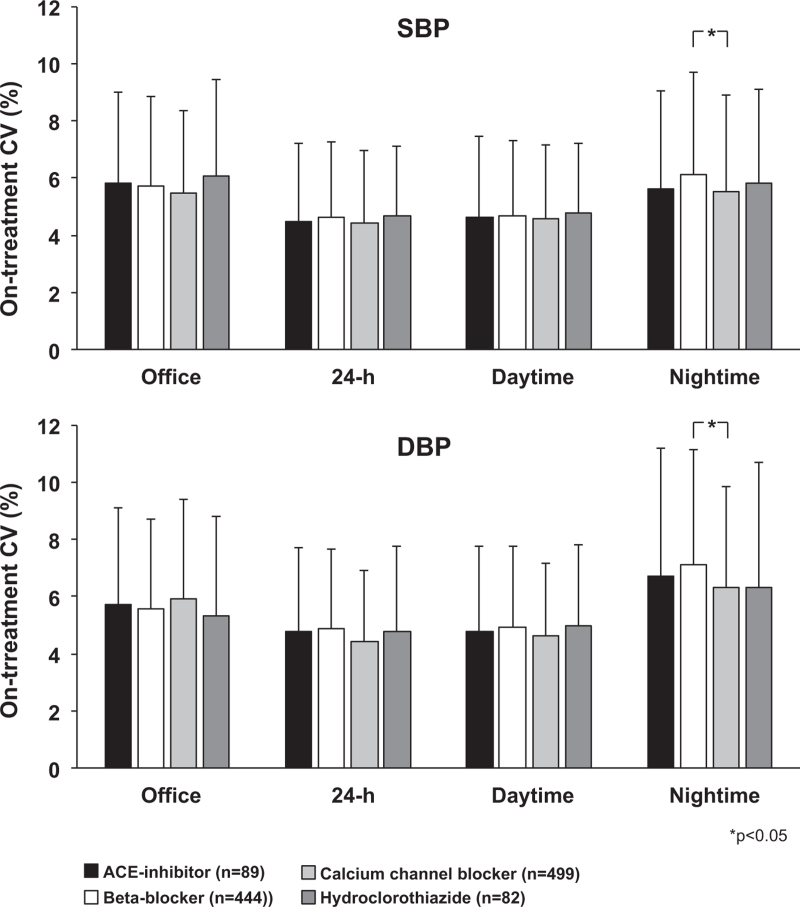
On-treatment visit-to-visit office, 24-h, day and night-time systolic (S) SBP and diastolic (D) BP coefficient of variations in patients under different antihypertensive treatments, i.e. a diuretic (hydroclorothiazide, *n* = 82), a calcium channel blocker (*n* = 499) a beta-blocker (*n* = 444) and an ACE-inhibitor (*n* = 89) Symbols as in preceding figures. With one exception values were not significantly different between groups.

As shown in Fig. 2, 24-h on-treatment visit-to-visit SBP-CV was less than office on-treatment SBP-CV the difference being statistically significant (−19.6%). The results were similar for on-treatment visit-to-visit DBP-CV (−18.7%). For both SBP and DBP the visit-to-visit CV was significantly and greater for the night-time than for the daytime (+20.0% and +28%, respectively) The results were similar when calculated according to patients’ sex, age, lower and higher baseline BP values and lower and higher achieved SBP or DBP values (Table 2, Supplemental Digital Content, 3 and Table 4, Supplemental Digital Content). With one exception (greater nighttime SBP-CV and DBP-CV under beta-blocker vs. calcium channel blocker treatment) office, 24-h, day and night-time visit-to-visit SBP-CV or DBP-CV did not differ significantly in differently treated groups (Fig. 3).

**FIGURE 4:**
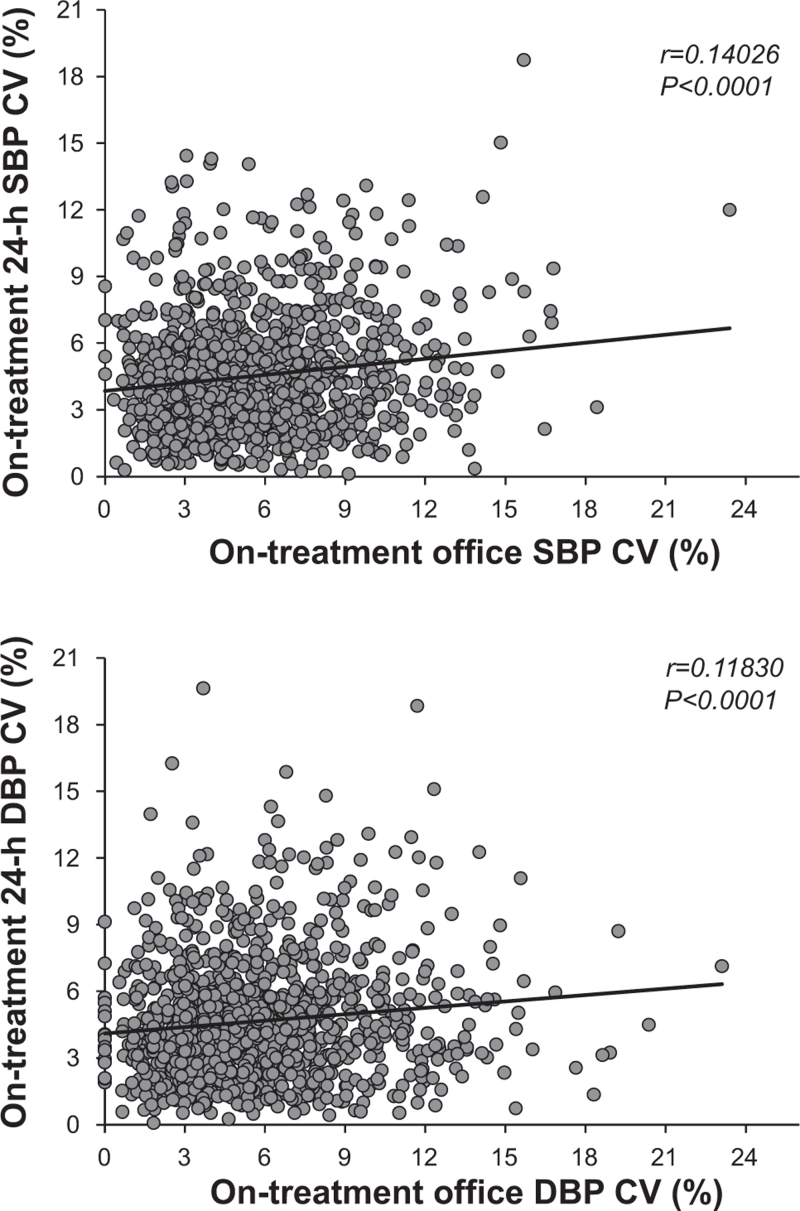
Relationship between on-treatment visit-to-visit office and 24-h SBP and DBP variability expressed as CV of the mean office and 24-h values during the treatment period. Symbols as in preceding figures and Table 1. *r*, coefficient of variation (Pearson).

As shown in Fig. 4, in the group as a whole there was a significant linear correlation between office and 24-h CVs, this being the case for both SBP and DBP. The correlation was significant in older and younger people, males and females, patients with higher and lower baseline BP and patients with higher and lower achieved BP (Table 2). However, as shown in Table 2, the correlation coefficients were invariably very low (approximately 0.1–0.2), indicating that only a small fraction of 24-h visit-to-visit BPV was predictable by office BPV, regardless of the sex, age and baseline or achieved office or ambulatory BP values. In the entire study population as well as in subgroups this fraction amounted to 1–4% of the visit-to-visit 24-h BPV (Table 5, Supplemental Digital Content).

**TABLE 2:** Correlations between office CV and 24-h on-treatment CV values in males and females as well as in subgroups with an age, baseline office SBP and DBP, and baseline 24-h SBP and DBP< and≥ the median value

	SBP		DBP	
Subgroups	*r*	*P*-value	*r*	*P*-value
Age <56 years	0.1366	0.0017	0.1517	0.0005
Age ≥56 years	0.1496	0.0003	0.0903	0.0290
Female	0.1965	<0.0001	0.1033	0.0182
Male	0.0904	0.0280	0.1328	0.0012
Baseline office SBP mean <161 mmHg	0.1254	0.0036	0.1626	0.0002
Baseline office SBP mean ≥161 mmHg	0.1617	<0.0001	0.0819	0.0499
Baseline office DBP mean <100 mmHg	0.1657	0.0002	0.1244	0.0051
Baseline office DBP mean ≥100 mmHg	0.1213	0.0028	0.1130	0.0053
Baseline 24-h SBP mean <139.3 mmHg	0.1429	0.0017	0.1240	0.0065
Baseline 24-h SBP mean ≥139.3 mmHg	0.1713	0.0002	0.1018	0.0258
Baseline 24-h DBP mean <87.6 mmHg	0.1235	0.0067	0.0972	0.0332
Baseline 24-h DBP mean≥87.6 mmHg	0.1867	<0.0001	0.1241	0.0065
On-treatment office SBP mean <140.25 mmHg	0.0799	0.0598	0.0713	0.0929
On-treatment office SBP mean ≥140.25 mmHg	0.1950	<0.0001	0.1671	<0.0001
On-treatment office DBP mean <85 mmHg	0.1023	0.0175	0.0984	0.0224
On-treatment office DBP mean ≥85 mmHg	0.1667	<0.0001	0.1386	0.0009
On-treatment 24-h SBP mean <130.8 mmHg	0.0933	0.0278	0.0870	0.0401
On-treatment 24-h SBP mean ≥130.9 mmHg	0.1805	<0.0001	0.151	0.0003
On-treatment 24-h DBP mean <80.5 mmHg	0.0699	0.0993	0.0676	0.1112
On-treatment 24-h DBP mean ≥80.5 mmHg	0.2045	<0.0001	0.1677	<0.0001

## DISCUSSION

Our analysis of the two trials that have made use of yearly office and ambulatory BP measurements during the antihypertensive treatment period provides a number of findings. One, confirming previous findings from one of the trials [14] on-treatment visit-to-visit 24-h BPV was smaller than visit-to-visit office BPV, this being the case for both SBP and DBP. Two, visit-to-visit 24-h BPV was lower than office BPV in different demographic subgroups (age and sex), subgroups with different baseline office or 24-h BP values, subgroups with different achieved office or 24-h BP values and subgroups under different pharmacological treatments. Three, in the group as a whole, as well as in the subgroups, the mean difference between 24-h BPV and office BPV was 19.6% (all patients) or greater (some subgroups), thereby never being quantitatively marginal. Finally, and most importantly, although visit-to-visit office and 24-h BPV correlated significantly both in the group as a whole and in most subgroups, the correlation coefficients were always very low (values approximately between 0.1 and 0.2) indicating that visit-to-visit office BPV predicted only a minute fraction (1–4%) of the visit-to-visit 24-h BP variations. These findings allow to conclude that in treated hypertensive patients 24-h mean BP across visits is noticeably more stable than office BP, independently on the treatment type, the demographic characteristics and the BP levels. This can presumably be explained by the fact that the multiple values provided by ambulatory BP favor stability of the mean values more than the few measurements provided by office BP. They also document, however, that visit-to-visit office BPV does nor reliably parallel the magnitude of 24-h BPV across visits, a small visit-to-visit office BPV in an individual patient being compatible with a large ambulatory BPV and vice versa. This means that it is impossible for the physician to predict the greater or lower consistency of ambulatory BP control across visits based on available office BP data. Although, the prognostic importance of visit-to-visit BPV has not been addressed by the present study (see below) and is presently unknown, this is likely to be a limitation of clinical relevance because 24-h BP has been repeatedly found to have a predictive ability for cardiovascular events greater than office BP [20–23].

Our study provides new findings on the ability of antihypertensive drug treatment to control night-time BP as well as on the consistency of treatment-dependent night-time BP control across visits. First, night-time BP control by treatment was considerably less than daytime BP control. Second, visit-to-visit night-time BPV was considerably greater than visit-to-visit daytime variability and in absolute values its magnitude was similar to visit-to visit office BPV. Third, persistence of night-time BP control across visits (i.e. control for all on-treatment visits) was much smaller for night-time than for daytime BP. Our study does not provide an explanation for these findings although it is conceivable that the poor intrinsic reproducibility of night-time BP reductions shown by several studies [24–26] and attributed to physiological factors (e.g. difference in depth and quality of sleep between nights) and/or to the variable influence of sleep apnea and other sleep disorders on night-time BP [27–29] play a role. Regardless of the factors involved, the lower ability of antihypertensive treatment to control night-time BP and to make this control consistent across visits is likely to reduce the contribution of night-time BP reduction to patient's protection by antihypertensive treatment, thus contributing to the residual cardiovascular risk that characterizes hypertensive patients [12,30].

Three other results of our study deserve a comment. One, our data show that both visit-to-visit office and 24-h BPV did not differ significantly between treatments based on a beta blocker, a calcium channel blocker, a converting enzyme inhibitor and a thiazide diuretic. This leads to the conclusion that, among major antihypertensive drugs, none is associated with any substantial advantage or disadvantage as far as BPV across visits is concerned. This is in contrast with the results of a meta-analysis of studies on office visit-to-visit BPV performed years ago [31], which reported antihypertensive medicaments to differently affect visit-to-visit office BPV. However what was measured in this meta-analysis was the variation of the office BP response to drug treatment between patients, an interindividual BP variability that has conceptually nothing to do with the smaller or greater stability of a BP response in a given patient. Two, given its superior prognostic significance [31–32] the greater stability of on-treatment 24-h BP should have favorable implications for patients’ protection during antihypertensive treatment. However our findings also show that, in line with what was reported by a meta-analysis of randomized trials almost 20 years ago [33], the number of patients achieving 24-h BP control is lower than the number of patients achieving office BP control and that this difference is particularly evident for persistent ambulatory BP control. This means that the greater 24-h BP stability between visits occurs at values that are more frequently above target than office BP values, which may neutralize its potentially favorable clinical effect. Three, visit-to-visit office, 24-h, daytime and night-time BPV were similar in patients with baseline or on-treatment BP < or ≥ the median value. Thus, the results of the present study are not affected by the initial office or out-of-office BP levels as well as by the greater or smaller office or out-of-office BP reduction with treatment.

Our study has strengths and limitations. The strengths are (i) the novelty of the results and their entire origin from within-patient comparisons and (ii) the standardization and quality of both office and ambulatory BP measurements, as inferred in the latter case from the high number of valid BP readings throughout the 24-h recording period and the study duration and (iii) the identical number of visits and visit intervals between office and ambulatory BP measurements. On the other hand, a limitation is that as commonly done, fewer BP readings were set for the night than for the daytime, possibly leading to some imbalance in the calculation of their mean and variability values. However, errors in the estimation of mean ambulatory BP values and variability have been reported with one or less than one reading per hour [34], not with the three-night hourly readings adopted in our studies; A second limitation is that, in our two trials, the overall rate of both office and 24-h BP control by treatment was rather low compared to other trials, presumably because in the protocol only one nonstudy drug could be added to the drug under study, limiting treatment to two rather than three drug combinations [12]. However, as mentioned above, the differences and correlations between visit-to-visit office and ambulatory BPV were not affected by the higher or lower on-treatment office or ambulatory BP, thereby being independent of on-treatment BP levels. Finally our results do not offer any information on the relationship of visit-to-visit 24-h BP variability with outcomes because both in ELSA and PHYLLIS the number of cardiovascular events (142 in ELSA and 6 in PHYLLIS) was too small for addressing this issue. Although the documented prognostic importance of ambulatory BP [7–12] suggests that its visit-to-visit variability and persistence under control are likely to be clinically relevant, this should be regarded as an important area for future research. This research should be extended to office and ambulatory visit-to-visit BPV in medical practice to determine the extent to which patient protection by treatment is adversely affected by inconsistency of office and out-of-office BP control, thus playing a role in the persistent position of hypertension as a major cause of death and disease in real life [35].

## ACKNOWLEDGEMENTS

Source of Funding: The original ELSA and Phyllis trials received financial support from Glaxo and Squibb, respectively. No Company financially supported the present study.

Disclosure: None.

### Conflicts of interest

There are no conflicts of interest.

## Supplementary Material
