# Supplementary material for: Comparison between visit-to-visit office and 24-h blood pressure variability in treated hypertensive patients
Source: J Hypertens. 2023 Oct 9;42(1):161–8. doi: 10.1097/HJH.0000000000003582 (PMC10712992; doi:10.1097/HJH.0000000000003582)
Supplement: Supplementary file 1 [file jhype-42-161-s001.docx]

**Supplementary Table S1** Map 24h: Percent (means ± SD) of valid ambulatory SBP readings in relation to the expected number of readings, 4 per hour for the day and 3 per hour for the night. Data are shown for the 24h, the day and the night at baseline and during treatment in all patients analyzed percent were calculated. T: treatment, ys: years; h: hour. Other symbols as in preceding Tables.

|  | 24-h | Day | Night |
| --- | --- | --- | --- |
| Baseline | 92.6±6.8 | 90.3±9.3 | 97.6±7.8 |
| T,1 ys | 91.6±7.7 | 89±9.9 | 96.3±9.8 |
| T, 2ys | 92.6±6.5 | 91±8.1 | 97.6±6.5 |
| T, 3-4ys | 92.8±6.4 | 91.5±7.5 | 97.5±6.1 |

**Supplementary Table S2** Average (±SD) coefficients of variation (CV) of the SBP or DBP mean values over the treatment period in all patients analyzed. Data are shown for patients < or ≥ median age and sex. Symbols as in preceding tables

|  | Age | |  | Sex | |  |
| --- | --- | --- | --- | --- | --- | --- |
|  | <56ys | ≥56 | p-value | Female | Male | p-value |
| Office SBP CV | 5.6±3.1 | 5.7±3.0 | 0.4068 | 5.9±3.3 | 5.4±2.8 | 0.0063 |
| 24-h SBP CV | 4.5±2.6 | 4.6±2.6 | 0.5281 | 4.6±2.5 | 4.5±2.6 | 0.4907 |
| Day SBP CV | 4.6±2.6 | 4.7±2.6 | 0.4973 | 4.7±2.5 | 4.6±2.7 | 0.7074 |
| Night SBP CV | 5.7±3.5 | 5.9±3.4 | 0.4736 | 5.9±3.5 | 5.7±3.4 | 0.2093 |
| Office DBP CV | 5.7±3.4 | 5.8±3.2 | 0.6140 | 5.7±3.4 | 5.7±3.3 | 0.8255 |
| 24-h DBP CV | 4.7±2.6 | 4.6±2.8 | 0.6922 | 4.7±2.7 | 4.7±2.7 | 0.9947 |
| Day DBP CV | 4.8±2.5 | 4.8±2.9 | 0.6303 | 4.8±2.7 | 4.8±2.7 | 0.7829 |
| Night DBP CV | 6.6±3.8 | 6.7±3.9 | 0.6339 | 6.7±4.0 | 6.6±3.8 | 0.6111 |

**Supplementary Table S3** Average (±SD) coefficients of variation (CV) of the SBP or DBP mean values over the treatment period in all patients analyzed. Data are shown for patients < or ≥ median baseline office BP, baseline mean 24-h BP, baseline mean day BP and baseline mean night BP. Symbols as in preceding tables

|  | Baseline Office SBP mean | |  | Baseline Office DBP mean | |  |
| --- | --- | --- | --- | --- | --- | --- |
|  | <161 mmHg | ≥161 mmHg | p-value | <100 mmHg | ≥100 mmHg | p-value |
| Office SBP CV | 5.6±3.1 | 5.7±3.0 | 0.5954 | 5.7±3.1 | 5.5±3.0 | 0.2699 |
| 24-h SBP CV | 4.6±2.6 | 4.5±2.5 | 0.5685 | 4.6±2.6 | 4.4±2.6 | 0.2469 |
| Day SBP CV | 4.7±2.7 | 4.6±2.5 | 0.3701 | 4.7±2.6 | 4.6±2.6 | 0.2590 |
| Night SBP CV | 5.8±3.4 | 5.9±3.5 | 0.6355 | 5.9±3.5 | 5.7±3.5 | 0.3652 |
| Office DBP CV | 5.6±3.3 | 5.8±3.4 | 0.2558 | 5.8±3.4 | 5.7±3.3 | 0.7483 |
| 24-h DBP CV | 4.8±2.7 | 4.5±2.7 | 0.0471 | 4.7±2.7 | 4.6±2.7 | 0.5722 |
| Day DBP CV | 5.0±2.8 | 4.6±2.6 | 0.0263 | 4.8±2.7 | 4.7±2.7 | 0.5213 |
| Night DBP CV | 6.6±3.9 | 6.7±3.9 | 0.7536 | 6.6±3.8 | 6.7±3.9 | 0.7431 |

|  | Baseline 24-h SBP mean | |  | Baseline 24-h DBP mean | |  |
| --- | --- | --- | --- | --- | --- | --- |
|  | <139.3 mmHg | ≥139.3 mmHg | p-value | <87.6 mmHg | ≥87.6 mmHg | p-value |
| Office SBP CV | 5.4±3.0 | 5.9±3.1 | 0.0141 | 5.6±3.0 | 5.7±3.1 | 0.4965 |
| 24-h SBP CV | 4.5±2.5 | 4.5±2.6 | 0.9061 | 4.4±2.4 | 4.6±2.7 | 0.3563 |
| Day SBP CV | 4.6±2.5 | 4.6±2.6 | 0.7909 | 4.6±2.5 | 4.7±2.7 | 0.3993 |
| Night SBP CV | 5.8±3.3 | 5.8±3.5 | 0.9889 | 5.7±3.2 | 5.8±3.5 | 0.4975 |
| Office DBP CV | 5.4±3.2 | 6.0±3.5 | 0.0088 | 5.7±3.3 | 5.7±3.4 | 0.8436 |
| 24-h DBP CV | 4.6±2.7 | 4.6±2.6 | 0.7910 | 4.5±2.7 | 4.7±2.7 | 0.2449 |
| Day DBP CV | 4.7±2.8 | 4.7±2.6 | 0.7679 | 4.6±2.7 | 4.8±2.7 | 0.4089 |
| Night DBP CV | 6.6±3.8 | 6.6±3.9 | 0.8871 | 6.5±3.8 | 6.7±3.9 | 0.3888 |

|  | Baseline Day SBP mean | |  | Baseline Day DBP mean | |  |
| --- | --- | --- | --- | --- | --- | --- |
|  | <143 mmHg | ≥143 mmHg | p-value | <90.9 mmHg | ≥90.9 mmHg | p-value |
| Office SBP CV | 5.4±3.0 | 5.9±3.1 | 0.0120 | 5.5±3.1 | 5.8±3.0 | 0.1483 |
| 24-h SBP CV | 4.5±2.5 | 4.5±2.6 | 0.7082 | 4.3±2.4 | 4.7±2.8 | 0.0471 |
| Day SBP CV | 4.6±2.6 | 4.6±2.6 | 0.8248 | 4.4±2.4 | 4.8±2.7 | 0.0404 |
| Night SBP CV | 5.8±3.3 | 5.8±3.4 | 0.8517 | 5.7±3.2 | 5.9±3.6 | 0.4223 |
| Office DBP CV | 5.3±3.0 | 6±3.6 | 0.0015 | 5.6±3.2 | 5.8±3.4 | 0.4732 |
| 24-h DBP CV | 4.7±2.7 | 4.5±2.6 | 0.2371 | 4.4±2.6 | 4.7±2.7 | 0.0890 |
| Day DBP CV | 4.8±2.8 | 4.6±2.6 | 0.2483 | 4.6±2.7 | 4.9±2.7 | 0.1208 |
| Night DBP CV | 6.6±3.8 | 6.6±3.8 | 0.8620 | 6.5±3.7 | 6.7±3.9 | 0.3226 |

|  | Baseline Night SBP mean | |  | Baseline Night DBP mean | |  |
| --- | --- | --- | --- | --- | --- | --- |
|  | <125.9 mmHg | ≥125.9 mmHg | p-value | <76.8 mmHg | ≥76.8 mmHg | p-value |
| Office SBP CV | 5.5±3.0 | 5.9±3.1 | 0.0478 | 5.5±3.0 | 5.8±3.1 | 0.1296 |
| 24-h SBP CV | 4.4±2.4 | 4.6±2.7 | 0.1009 | 4.5±2.4 | 4.5±2.7 | 0.9543 |
| Day SBP CV | 4.5±2.4 | 4.8±2.7 | 0.0780 | 4.6±2.4 | 4.6±2.7 | 0.8614 |
| Night SBP CV | 5.7±3.3 | 5.9±3.5 | 0.3167 | 5.8±3.3 | 5.8±3.5 | 0.9171 |
| Office DBP CV | 5.5±3.3 | 5.8±3.4 | 0.1921 | 5.7±3.4 | 5.7±3.3 | 0.8522 |
| 24-h DBP CV | 4.6±2.6 | 4.6±2.7 | 0.6695 | 4.6±2.6 | 4.6±2.7 | 0.7709 |
| Day DBP CV | 4.7±2.7 | 4.8±2.7 | 0.6413 | 4.7±2.7 | 4.7±2.7 | 0.9068 |
| Night DBP CV | 6.6±3.8 | 6.7±3.9 | 0.7387 | 6.7±3.7 | 6.6±4.0 | 0.8166 |

**Supplementary Table S4** Average (±SD) coefficients of variation (CV) of the SBP or DBP mean values over the treatment period in all patients analyzed. Data are shown for patients < or ≥ median on-treatment office BP, on-treatment mean 24-h BP. Symbols as in preceding tables

|  | On-treat Office SBP mean | |  | On-treat Office DBP mean | |  |
| --- | --- | --- | --- | --- | --- | --- |
|  | <140.25 mmHg | ≥140.25 mmHg | p-value | <85 mmHg | ≥85 mmHg | p-value |
| Office SBP CV | 5.5±2.9 | 5.8±3.2 | 0.0924 | 5.3±2.8 | 5.9±3.2 | 0.0018 |
| 24-h SBP CV | 4.5±2.5 | 4.6±2.6 | 0.4172 | 4.4±2.5 | 4.7±2.7 | 0.1130 |
| Day SBP CV | 4.6±2.5 | 4.7±2.7 | 0.2700 | 4.6±2.5 | 4.7±2.7 | 0.2713 |
| Night SBP CV | 5.8±3.5 | 5.8±3.4 | 0.7023 | 5.6±3.4 | 6±3.5 | 0.0259 |
| Office DBP CV | 5.6±3.3 | 5.8±3.3 | 0.2817 | 5.7±3.5 | 5.7±3.2 | 0.8584 |
| 24-h DBP CV | 4.7±2.7 | 4.6±2.7 | 0.5131 | 4.6±2.7 | 4.7±2.7 | 0.6595 |
| Day DBP CV | 4.8±2.7 | 4.8±2.7 | 0.6546 | 4.8±2.7 | 4.8±2.7 | 0.9308 |
| Night DBP CV | 6.7±4.1 | 6.6±3.6 | 0.8038 | 6.6±4 | 6.8±3.8 | 0.4036 |

|  | On-treat 24h SBP mean | |  | On-treat 24h DBP mean | |  |
| --- | --- | --- | --- | --- | --- | --- |
|  | <130.8 mmHg | ≥130.8 mmHg | p-value | <80.5 mmHg | ≥80.5 mmHg | p-value |
| Office SBP CV | 5.4±2.9 | 5.9±3.2 | 0.0147 | 5.5±3 | 5.8±3.1 | 0.1676 |
| 24-h SBP CV | 4.4±2.5 | 4.7±2.6 | 0.0959 | 4.4±2.5 | 4.7±2.7 | 0.0760 |
| Day SBP CV | 4.5±2.6 | 4.8±2.6 | 0.1039 | 4.5±2.5 | 4.8±2.7 | 0.1669 |
| Night SBP CV | 5.7±3.2 | 5.9±3.7 | 0.4161 | 5.7±3.3 | 5.9±3.6 | 0.3277 |
| Office DBP CV | 5.5±3.2 | 6±3.4 | 0.0083 | 5.6±3.3 | 5.8±3.3 | 0.3380 |
| 24-h DBP CV | 4.7±2.7 | 4.6±2.7 | 0.6076 | 4.6±2.7 | 4.7±2.7 | 0.6137 |
| Day DBP CV | 4.8±2.8 | 4.7±2.7 | 0.5632 | 4.8±2.7 | 4.8±2.7 | 0.9024 |
| Night DBP CV | 6.7±3.9 | 6.7±3.9 | 0.9776 | 6.6±3.9 | 6.7±3.9 | 0.6977 |

**Supplementary Table S5** Percentage of 24-h SBP-CV or DBP-CV accounted for by office SBP-CV or DBP-CV, respectively in all patients and in patients subgroups of Supplementary Tables 2, 3 and 4.Percentages were derived from the square elevation(R^2^). of the correlation coefficients between SBP-CV or DBP-CV and 24-h SBP-CV or DBP-CV. Abbreviations as in preceding Tables

|  | **SBP** | | **DBP** | |
| --- | --- | --- | --- | --- |
| Subgroups | **R^2^** | p-value | **R^2^** | p-value |
| ALL patients | 1.97% | <0.0001 | 1.40% | 0.00% |
| Age <56ys | 1.87% | 0.0017 | 2.30% | 0.0005 |
| Age ≥56ys | 2.24% | 0.0003 | 0.82% | 0.029 |
| Female | 3.86% | <.0001 | 1.07% | 0.0182 |
| Male | 0.82% | 0.028 | 1.76% | 0.0012 |
| Baseline Office SBP mean<161 mmHg | 1.57% | 0.0036 | 2.64% | 0.0002 |
| Baseline Office SBP mean≥161 mmHg | 2.61% | <.0001 | 0.67% | 0.0499 |
| Baseline Office DBP mean<100 mmHg | 2.75% | 0.0002 | 1.55% | 0.0051 |
| Baseline Office DBP mean≥100 mmHg | 1.47% | 0.0028 | 1.28% | 0.0053 |
| Baseline 24-h SBP mean<139.3 mmHg | 2.04% | 0.0017 | 1.54% | 0.0065 |
| Baseline 24-h SBP mean≥139.3 mmHg | 2.93% | 0.0002 | 1.04% | 0.0258 |
| Baseline 24-h DBP mean<87.6 mmHg | 1.53% | 0.0067 | 0.94% | 0.0332 |
| Baseline 24-h DBP mean≥87.6mmHg | 3.49% | <.0001 | 1.54% | 0.0065 |
| On-treatment Office SBP mean<140.25 mmHg | 0.64% | 0.0598 | 0.51% | 0.0929 |
| On-treatment Office SBP mean≥140.25 mmHg | 3.80% | <.0001 | 2.79% | <.0001 |
| On-treatment Office DBP mean<85 mmHg | 1.05% | 0.0175 | 0.97% | 0.0224 |
| On-treatment Office DBP mean≥85 mmHg | 2.78% | <.0001 | 1.92% | 0.0009 |
| On-treatment 24-h SBP mean<130.8 mmHg | 0.87% | 0.0278 | 0.76% | 0.0401 |
| On-treatment 24-h SBP mean≥130.9 mmHg | 3.26% | <.0001 | 2.28% | 0.0003 |
| On-treatment 24-h DBP mean<80.5 mmHg | 0.49% | 0.0993 | 0.46% | 0.1112 |
| On-treatment 24-h DBP mean≥80.5mmHg | 4.18% | <.0001 | 2.81% | <.0001 |
